# When time turns the tide: the interactive effects of ammonium and warming during the larval stage on the resulting adult frogs

**DOI:** 10.1186/s12983-025-00585-z

**Published:** 2025-11-13

**Authors:** Francisco Javier Zamora-Camacho, Pedro Aragón

**Affiliations:** 1 Department of Biogeography and Global Change, Museo Nacional de Ciencias Naturales (MNCN-CSIC), C/José Gutiérrez Abascal 2, 28006 Madrid, Spain; 2 Department of Biology of Organisms and Systems, University of Oviedo, C/Valentín Andrés Álvarez S/N, 33071 Oviedo, Spain

**Keywords:** Anuran, Contamination, Global change, Global warming, *Pelophylax perezi*.

## Abstract

**Background:**

Two central elements of the global change are a rise in average temperatures and the contamination of natural habitats, among others, with agricultural fertilizers, which can stress living beings. Avoidance of these stressors is often impossible for animals confined in certain habitats, such as pond-dwelling tadpoles, which can indeed select less stressful microhabitats after metamorphosis. However, the stressors faced during the larval stage may have long-term repercussions.

**Methods:**

In this work, we reared tadpoles in either heated or non-heated tanks, in each case either exposed or not to ammonium contamination. The resultant metamorphs were reared, released from such stressors, until adulthood, when their body size, body condition and locomotor performance were gauged.

**Results:**

Whereas larvae reared in non-heated tanks responded to ammonium with a lower body size as adults, in accordance with previous experiments, the opposite was true for those reared in heated tanks. Body condition was greater in ammonium-exposed individuals, in which locomotor performance was reduced, as compared with non-ammonium-exposed conspecifics.

**Conclusions:**

Greater body size in adults in heated water as a response to ammonium could be a consequence of temperature mediating detoxifying metabolic routes of tadpoles. Better body condition and worse locomotor performance in ammonium-exposed individuals could reflect a prioritization of the storage of resources to the detriment of locomotion in ammonium-exposed individuals, or a limitation in the ability to optimize locomotion but not storage of resources.

**Supplementary Information:**

The online version contains supplementary material available at 10.1186/s12983-025-00585-z.

## Introduction

One of the greatest scientific challenges of the last decades, involving various fields of knowledge, is the multi-dimensional global change planet Earth is undergoing [1, 2]. The ever-expanding human population, along with increasing per-capita consumption habits, are exerting enormous pressure on the ecological balance worldwide in multiple forms [3]. These factors in combination are primarily leading to an escalation in cropland extension [4] in conjunction with an increment in intensive agricultural techniques [5]. The immediate outcome is a major change in land use [6] and the input of massive amounts of agrochemicals of different kinds [7], particularly fertilizers [8], into the environment. A portion of these fertilizers, especially nitrogenous compounds, are injected into the atmosphere in the form of greenhouse effect gases [9]. In addition, most human activities, especially but not limited to the management of energy resources, industry and transportation, are also releasing volumes of greenhouse effect gases, which altogether underlie an alarming global warming [10].

The effects that those stressors, either alone or in combination, may have on organisms are complex to disentangle. To begin with, fertilizers disrupt physiological functions of animals [11], while potentiating primary production [12]. The increase in food resources triggered by the latter could offset and obscure the negative consequences of the former to a certain extent [13]. However, fertilizers, especially nitrogenous compounds, may unbalance nutrient cycles [14] and, in aquatic environments, lead to acidification, eutrophication and even toxicity [15]. In turn, the global warming has also triggered changes whose consequences are intricate to predict, which span from physiological [16], morphological [17], ontogenetic [18], chorological [19] to even phenological [20]. The synergy between nitrogenous compounds and high temperatures can further exacerbate primary production [21] and potentiate the performance [22] and reproduction [23] of certain consumers, leading to unbalanced ecosystem function, and promoting oxidative stress [24] and even mortality [25] at the individual level. The climate change in general, and the global warming in particular, are known to modify the responses of organisms to nitrogenous compounds in terrestrial and freshwater environments [26], which warrants further research on the ecological consequences of the interaction between these global change components.

The ability of animals to withstand these stressors can vary intrinsically, both intraspecifically [27, 28] and among taxa [29, 30], and extrinsically, for instance depending on the intensity and duration of the exposure [31]. Typically, animals in contact with stressors actively avoid or minimize the exposure to them [32], optimizing their use of space by selecting microhabitats free from unsuitable temperatures [33] or excessive pollutants [34]. However, stressors may be inescapable under certain circumstances, such as in the case of pervasive stressors or strongly compartmented habitats that animals cannot abandon [35]. Moreover, the success of avoidance after exposure can be restricted if the stressor in question has long-term effects which are apparent after release [36].

Amphibians are fitting model organisms to study those phenomena for various reasons. In the first place, amphibians are highly sensitive to chemicals, especially those capable of traversing their permeable skins [37], such as inorganic nitrogenous compounds [38]. Among these, ammonium is a particularly frequent contaminant derived from crop fertilization [39], and has contrasted negative effects on amphibians [40–42]. Moreover, as ectotherms, these animals are highly dependent on environmental temperatures to attain their optimal thermal ranges [43], within which traits such as growth, development [44], locomotion [45] and survival [46] are maximized. Consequently, an alteration of environmental temperatures can inflict major consequences on the ecology and physiology of amphibians [47] and exacerbate the negative consequences of pollutants [48]. Amphibians in their aquatic larval stages are confined within the water bodies they inhabit, thus being forced to face the thermal and chemical conditions in them [49]. Contrastingly, amphibians in the terrestrial stage can and do avoid locations where temperature [50] or pollution [51] are detrimental. Nonetheless, the conditions experienced during the larval stage may have consequences that endure long after the metamorphosis, and therefore after the release from said conditions [52, 53].

Iberian green frog (*Pelophylax perezi*; López-Seoane, 1885) tadpoles were chronically exposed to either 1) increased temperature, 2) a sublethal dose of ammonium, 3) both simultaneously, or 4) none of them, in a 2 × 2 experimental design [54]. They showed overriding effects of increased temperature, which masked any potential consequence of ammonium: an increase in temperature resulted in larger and faster larvae, but which metamorphosed into smaller froglets with poorer locomotor performance [54]. By contrast, in experiments where temperature was not manipulated, ammonium did exert negative effects on larvae [42], metamorphs and adults [55]. In this work, we investigate the long-term carryover effects of such a treatment (manipulated temperature and/or exposure to ammonium throughout the larval stage) on the resulting adult frogs, after being released from any alteration of temperature or contamination of any kind for 14 months. Given that artificially raised temperature produced smaller metamorphs with worse locomotor performance, we predict that these negative consequences will persist until adulthood.

## Materials and methods

### Study species

*Pelophylax perezi* (Anura: Ranidae) is a medium-sized frog (the average snout-vent length [SVL] in this sample was 51.25 mm) widely distributed in the southwest of Europe. It can be found in large numbers in a variety of habitats, albeit always in or next to diverse types of water bodies, which can range from pristine to brackish and even polluted. These frogs reproduce in the late spring or the early summer, when the females lay numerous egg masses along each mating season, each containing from some dozens to even thousands of eggs. The larval period spans from two to four months in most cases. The larvae feed on various sources of organic matter, mostly plant carrion, whereas the adults are mainly insectivore. In turn, these frogs are frequently preyed on by snakes, mammals and birds, among others, from which they flee by means of vigorous leaps towards a refuge or, most commonly, the water [56, 57].

### Animal capture and handling

In May 2023, a total of 10 males and 10 females were captured in two different ponds in Pinares de Cartaya (SW Iberian Peninsula, 37° 20′ N, 7° 09′ W), a *Pinus pinea* grove with an understory of *Cistus ladanifer*, *Pistacia lentiscus* and *Rosmarinus officinalis*. These frogs were housed together in an outdoor seminatural enclosure of 6 × 6 m, with a 1 m-high brick wall supporting a 1 m-high, 5 mm-size rustproof metal mesh which also covered the roof. Thus, the frogs were not able to escape the enclosure, nor were predators able to enter it, whereas the small invertebrates the frogs feed on could easily go through the mesh, providing the frogs with abundant food. We regularly made sure the abundance of invertebrates was enough to feed the frogs. The enclosure possessed abundant refuges in the form of cork tree bark, as well as an 11 m^2^, 50 cm deep pond where frogs could reproduce (see Supplementary Material in [58]). Once the frogs were housed in this enclosure, the pond was searched for egg masses every day, and each egg mass located was transported to the laboratory. When 19 egg masses had been collected, the adult frogs were released at their capture site.

Each egg mass was then spread in a tray, where we randomly selected 15 individual eggs from different areas within the tray in no particular order to be transferred to a plastic tank (19 × 38 x 27 cm) containing 6 L of untreated water. Each tank was then randomly allotted to one among four possible combinations of a 2 × 2 experimental design with two factors (thermal treatment and ammonium treatment), each with two levels. Concerning the thermal treatment, in heated tanks we added a 50 W submergible water heater (Marina Mini), continuously connected, whereas in non-heated tanks no heater was added, so that water was at room temperature. Concerning the ammonium treatment, in tanks with ammonium we added an approximate 178.87 mg of 99.7% pure NH_4_Cl, so that the ammonium (NH_4_^+^) concentration was of 10 mg/L. A concentration of 13.5 mg NH_4_^+^/L is known to provoke a mortality rate of 70% after a 21-day exposure in *P. perezi* tadpoles [59].

We made use of a slightly lower concentration in order to avoid such mortality, while still triggering sublethal effects [42]. Moreover, this concentration is frequent in freshwater bodies in Spain [41, 60]. In tanks without ammonium, no NH_4_Cl was added. In total, there were 5 tanks heated with ammonium, 5 heated without ammonium, 5 non-heated with ammonium, and 4 non-heated without ammonium. The tanks were maintained on laboratory shelves, with a window letting natural light in for the adjustment of circadian rhythms. Room temperature was not controlled, and fluctuated with the weather. The water was changed twice a week, and each time the heater was returned to the heated tanks, the same amount of ammonium described was added to the tanks with ammonium, and the position of each tank within the selves was modified, to prevent the tadpoles from being chronically affected by potential subtle differences in light and temperature caused by such position. Every time we changed the water, until the first metamorph emerged, we measured water temperature with a thermometer (OcioDual SKU: 80,765; accuracy 0.1 ºC). We also measured the concentration of ammonium with an Ammonia HR Checker (Hanna Instruments, HI733; accuracy 0.1 mg/L) twice a week in a different subsample of tanks each time. Therefore, we could ensure that heated tanks had higher temperature than non-heated tanks, and that tanks with ammonium had a greater concentration of NH_4_^+^ than tanks without ammonium. The statistical analyses demonstrating that water was hotter in heated tanks, and that ammonium concentration was greater in ammonium-supplemented tanks, can be found in a previous study on the same individuals but at the larval stage [54], as this research makes part of a long-term project. Besides, the mean values of temperature and ammonium concentration in each measurement made are provided as Supplementary Material (Table S1). We fed tadpoles boiled spinach ad libitum.

Tadpoles were maintained as described throughout the larval period until metamorphosis, along July 2023. Tadpole mortality was checked twice a week. At that point, 50 metamorphs reared in each combination of thermal and ammonium treatment were selected in a quasi-random way, balancing the number of individuals extracted from each tank. Each group of 50 metamorphs was then transported to one of four adjacent semi-natural outdoor enclosures whose characteristics were the same as the one where the parentals were housed for reproduction (which have been described above), and reared until adulthood under similar conditions, released from any contamination or alteration of temperature or any other variable. Note that the metamorphs could not be individually marked, as their small size made the methods available (mainly, toe clipping, microchips, and visible implant elastomer) unsuitable or too invasive to guarantee survival. Thus, pooling the froglets separately in the enclosures according to the treatment they had been subjected to as larvae was the only way to be sure of the treatment experienced by a given adult frog when it was a tadpole. However, the fact that all frogs in each enclosure had undergone the same treatment makes it impossible to disentangle any potential effect of the factor “enclosure”. Nevertheless, the four enclosures had the exact same characteristics (see above), and they were adjacent to each other, so all the conditions experienced by the frogs during their time in the enclosures were exactly the same.

In September 2024, when they had reached sexual maturity, the survivors (39 females and 37 males; non-heated with ammonium: 8 females and 8 males; non-heated without ammonium: 12 females and 7 males; heated with ammonium 9 females and 17 males; heated without ammonium: 10 females and 5 males) were captured and transported to the laboratory. After a minimum of 20 min for acclimation to the laboratory room temperature (around 26 °C), we measured jumping distance on a horizontal metallic arena (200 × 90 cm). Each frog was individually released at one corner, and a magnet was placed right behind its urostyle. Then, we stimulated the frog to jump forward by prodding its hindquarters, and placed another magnet behind the urostyle where it landed. The use of magnets as landmarks on a metallic surface prevented the frog from removing or misplacing them when jumping. This process was repeated five times. Next, we measured the distance between consecutive magnets, equaling jumping distance, with a ruler (accuracy: 1 mm), and recorded the longest jump of each individual as its jumping distance. The arena was thoroughly rinsed after every trial.

We sexed the individuals based on the presence of vocal sacs in the mouth commissures and nuptial pads in the forelimbs, which are traits of males but not females [57]. Then, we measured their SVL with a ruler (accuracy: 1 mm) and their body mass with a digital scale (Gram FC; accuracy: 0.1 g), after gently removing any excess water with a disposable piece of absorbing paper. SVL informs about cumulative growth during the individual’s life, and body mass reflects the amount of matter the individual integrates at a given point, whereas the proportion of that matter that corresponds to energy reserves needs to be estimated by means of body condition indices such as the scaled mass index, which has proven to reliably correlate with the said reserves [61]. Therefore, we also calculated the SMI of each individual as per the following formula:

$${\text{SMI }} = W_{i} \left[ {\frac{{L_{0} }}{{L_{i} }}} \right]^{{b_{SMA} }}$$

where *W*_i_ and *L*_i_ represent the body mass and the SVL respectively of a particular individual, *L*_0_ represents the mean SVL of the sample, and ${b}_{SMA}$ (2.875 in this case) is the slope of a standardized major axis regression, obtained here as the ratio between the slope from the ordinary least squares regression of body mass on snout-vent length (both ln-transformed) by the Pearson’s correlation coefficient, r [62]. The metamorphs that were not used, and the adult frogs after the measurements, were released as soon as possible at their progenitors’ capture sites.

### Statistics

In the first place, we checked differences in survivorship with a general linear model linked to a logit function for data with a binomial distribution, using the function *glm* in the package “*stats*” in the R environment [63], where survival was the response variable, and thermal treatment, ammonium treatment and their interactions were included as fixed factors. Then, we conducted a series of general linear models where thermal treatment, ammonium treatment, sex and their interactions were fixed factors, and either SVL, body mass, SMI or jumping distance were response variables. Tukey post-hoc tests were applied on significant interactions (presented as Supplementary Material). The final models after applying stepwise backward selection equated to the full models, so we report the latter here for simplicity. The assumptions of homoscedasticity and residual normality were checked before applying parametric statistics [64]. Body mass was square-root transformed to meet the assumption of homoscedasticity. Bonferroni correction was applied, so the significance threshold was set at *P* = 0.0125.

## Results

### Survival

**Fig. 1:**
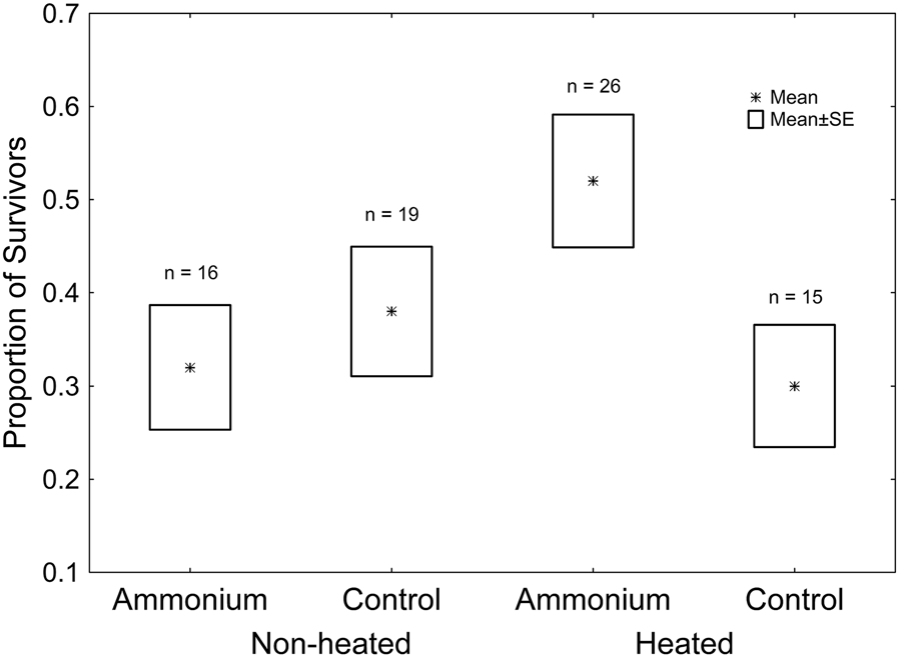
– Effects of ammonium and thermal treatment on adult frog survivorship. Asterisks represent mean values. Boxes represent standard errors

The effects of thermal treatment (*Χ*^2^_1, 196_ = 4.137; *P* = 0.042; Fig. 1), ammonium treatment (*Χ*^2^_1, 196_ = 5.051; *P* = 0.025; Fig. 1), and the thermal treatment*ammonium treatment interaction (*Χ*^2^_1, 196_ = 4.081; *P* = 0.043; Fig. 1), were significant, according to which survivorship was greater in adult frogs reared in heated water with ammonium. However, according to the Tukey post-hoc test performed on that interaction, no pairwise comparison was significant (in all cases, *P* > 0.110).

### Snout-vent length

**Fig. 2:**
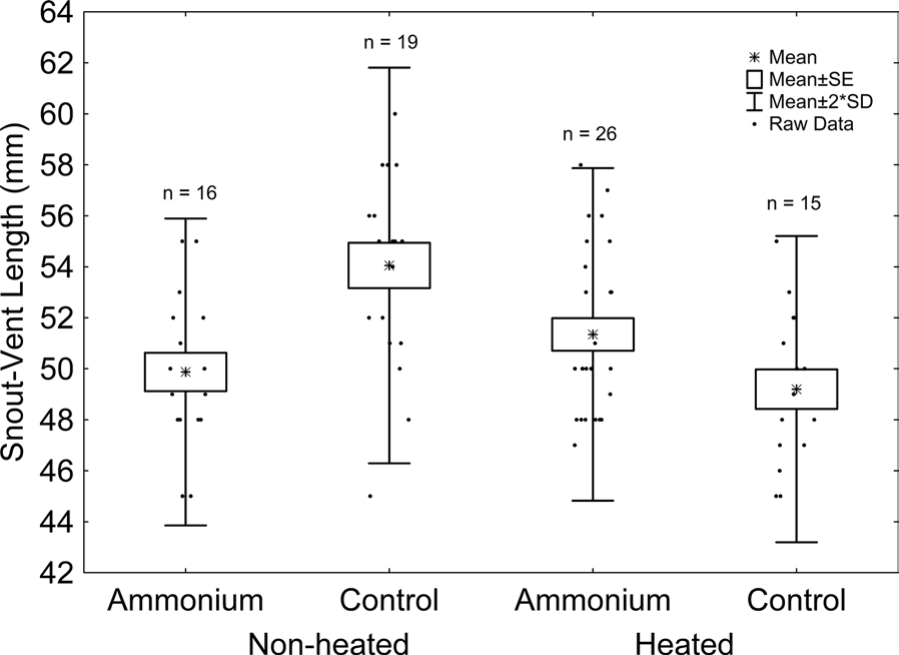
– Effects of ammonium and thermal treatment on frog SVL. Asterisks represent mean values. Boxes represent standard errors. Whiskers represent two times the standard deviation. Dots represent raw data. Sample sizes are indicated

The effects of thermal treatment, sex, and the thermal treatment*ammonium treatment interaction were significant (Table 1). Females were longer than males (mean ± standard error [mm]; females [n = 39]: 53.14 ± 0.40; males [n = 37]: 48.79 ± 0.45). According to the thermal treatment*ammonium treatment interaction, tadpoles reared in non-heated tanks grew into longer adult frogs in the absence of ammonium, whereas the opposite is true for those reared in heated tanks (Fig. 2, Table S2).

**Table 1:** – *F*-values of the full models testing the effects of thermal treatment, ammonium, sex, and their interactions on SVL, body mass, hindlimb length, SMI and jumping distance of adult frogs. Degrees of freedom were 1 and 68. Symbols indicate: ns = non-significant; * = *P* < 0.05; ** = *P* < 0.01; *** = *P* < 0.001. Significant results are in bold

	Snout-vent-length	Body mass	Scaled mass index	Jumping distance
Thermal treatment	4.03*	1.97^ ns^	0.47^ ns^	0.05^ ns^
Ammonium	0.01^ ns^	1.71^ ns^	6.11*	10.61**
Sex	52.20***	23.73***	10.30**	0.03^ ns^
Thermal Treatment*ammonium	33.06***	37.15***	3.00^ ns^	0.01^ ns^
Thermal treatment*sex	< 0.01^ ns^	0.47^ ns^	1.41^ ns^	2.02^ ns^
Ammonium*sex	0.52^ ns^	0.14^ ns^	0.06^ ns^	0.38^ ns^
Thermal Treatment*ammonium*sex	3.80^ ns^	3.94^ ns^	0.20^ ns^	2.99^ ns^

### Body mass

**Fig. 3:**
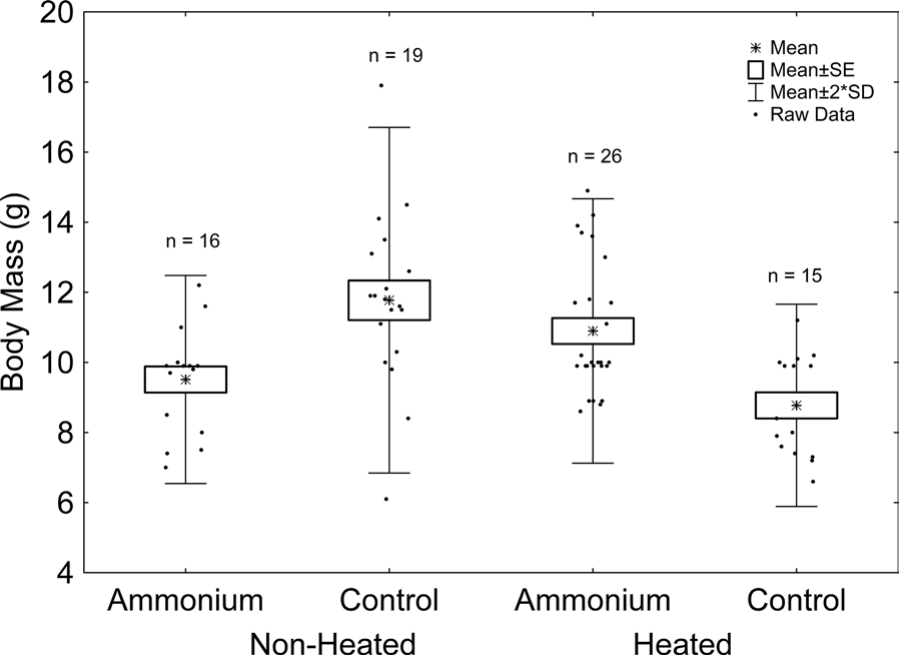
– Effects of ammonium and thermal treatment on frog body mass. Asterisks represent mean values. Boxes represent standard errors. Whiskers represent two times the standard deviation. Dots represent raw data. Sample sizes are indicated

The effects of sex and the thermal treatment*ammonium treatment interaction were significant (Table 1). Females were heavier than males (mean ± standard error [g]; females [n = 39]: 11.02 ± 0.20; males [n = 37]: 9.18 ± 0.21; back-transformed data; Table 1). According to the thermal treatment*ammonium treatment interaction, tadpoles reared in non-heated tanks grew into more massive adult frogs in the absence of ammonium, whereas the opposite is true for those reared in heated tanks (Fig. 3, Table S3).

### Scaled mass index

The SMI was significantly greater in males (mean ± standard error; females [n = 39]: 9.93 ± 0.13; males [n = 37]: 10.57 ± 0.15; Table 1) and in individuals reared in the presence of ammonium (mean ± standard error; with [n = 42]: 10.50 ± 0.13; without [n = 34]: 10.01 ± 0.15; Table 1).

### Jumping distance

Individuals reared in the absence of ammonium jumped significantly further (mean ± standard error; with [n = 42]: 38.38 ± 2.11; without [n = 34]: 48.69 ± 2.36; Table 1).

## Discussion

These results evidence that exposure to ammonium and increased temperature during the larval stage of these frogs have long-term repercussions that resonate up to their adult stage, despite manipulation release after metamorphosis. The only difference detected on metamorphs subjected to the same treatment was a greater body size in individuals reared in non-heated tanks, although as larvae they were smaller, with no effect of ammonium [54]. However, for adult frogs reared in non-heated tanks as tadpoles, we detected negative effects of ammonium on body size. One potential cause for this discrepancy could be the impossibility to sex the metamorphs. Adult females were larger than males, which coincides with previous studies on this species [57] and is a general trend in anurans [65]. If such sexual differences in body size manifest themselves at metamorphosis, or even in larvae, not controlling for sex could yield inaccurate results. Nonetheless, if that was not the case, then the negative effects of ammonium on growth were delayed until after the metamorphosis, thus after the exposure to it had finished, as observed in other pollutants affecting other amphibians (reviewed in [66]). In any case, a similar pattern was found in experiments performed in previous years, where tadpoles of this species were exposed to ammonium following the same protocol here, but where temperature was not manipulated: larvae were larger in the presence of ammonium [42], and no effect of ammonium on body size was patent at metamorphosis, but growth was reduced in adults once sex was controlled for [55].

Nonetheless, this negative effect of ammonium on body size was reversed in frogs reared in heated tanks during the larval stage. In these individuals, exposure to ammonium during the larval stage led to larger body sizes in adults. Interestingly, heated tanks produced larger larvae which, in turn, developed into smaller metamorphs after a shorter larval period, without an effect of ammonium [54]. The faster growth during the larval stage along with the shortened larval period may indicate accelerated metabolic processes promoted by higher temperature, which is common in ectotherms [67]. Certain detoxifying enzymes are known to be activated in greater amounts at high temperatures in some tadpoles [68], which could eliminate not only the short- but also the long-term negative effects of ammonium seen in frogs reared in non-heated tanks as larvae. Nevertheless, this potential explanation would suffice to clarify the absence of an effect of ammonium in these individuals, but not the positive impact on growth we observed. In any case, on average, these results challenge the tenet that size of froglets at metamorphosis predicts the size of the subsequent adults, which has been supported by some studies [69, 70] but not by others [55, 71]. It should also be noted that, aligned with this finding, survivorship was greater in ammonium-exposed individuals reared in heated water than in every other group, but the absence of statistical support for this difference in the pertinent post-hoc test calls for caution in the interpretation of such a result.

Reproductive success is usually favored in larger anurans, both in males [72] and females [73]. Therefore, reproduction could be hampered in frogs exposed to ammonium during the larval stage in colder water, but such an effect could be reversed in hotter environments. Body condition is also positively correlated with reproductive success in male [74] and female anurans [75]. Irrespective of temperature, tadpoles reared in the presence of ammonium turned into adults with greater SMI scores. Similar results have been found in previous experiments on this species [55]. Investment in reproduction is expected to be prioritized under circumstances capable of reducing lifespan, which could potentiate an efficient early reproduction if subsequent reproductive events are unlikely [76]. Accordingly, natterjack toads (*Epidalea calamita*) from agrosystems, intensively human-altered and contaminated areas, live shorter but invest more in reproduction than natural-habitat conspecifics [77]. However, we did not quantify reproduction, which makes these assertions speculative. Exposure to ammonium had strong negative effects on tadpole survival [54], but the question whether adult lifespan would also be shorter remains unanswered.

However, locomotor performance was impaired in frogs reared in the presence of ammonium, regardless of temperature. Locomotor performance does enhance survival in anurans [53] as well as other taxa [78–80]. Therefore, even if our data do not support greater intrinsic mortality in post-metamorphic frogs subjected to ammonium contamination during the larval stage, reduced locomotor performance could make them more susceptible to predators [81]. It should be noted that predators were purposefully excluded from the enclosures where the frogs were reared until adulthood, so that they were not subjected to predation pressure. Locomotor performance is also considered to improve reproductive success in multiple taxa [82], which could place frogs reared in the presence of ammonium at a reproductive disadvantage. However, this would contravene the alleged positive effect of body condition on reproduction, which is greater in ammonium-reared frogs. Nonetheless, the mechanisms by which locomotion improves reproduction in other animals, usually territorial agonistic encounters or mate persecution [83], might be irrelevant in anurans, which resort to a lekking system based on choruses of males that attract females [84]. In any case, our inferences concerning the potential effects of an exposure to ammonium during the larval stage on the reproductive success of adult frogs are highly speculative. What is apparent is the fact that ammonium-exposed individuals seem to prioritize the accumulation of resources, perhaps to the detriment of locomotor performance, or else, exposure to ammonium could be limiting the ability of these frogs to optimize locomotion, but not the accumulation of resources.

In metamorphs, the presence of ammonium did not affect locomotor performance, but jumping distance was greater in froglets reared in non-heated tanks [54]. Again, this reproduces the pattern found in a previous experiment manipulating ammonium but not temperature [55]. As in the case of body size, the overriding effects of temperature at the larval and metamorphic stages, up to a point in which ammonium was of negligible consequences [54], give way to stronger long-term effects of ammonium than temperature in adults.

It should be noted that these results could be biased if sex-dependent mortality may have led to differential survivorship between the sexes among treatment combinations [85], as the non-surviving individuals could obviously not be measured. However, the sex ratio was nearly 50% (37 males and 39 females), which does not support this possibility. Likewise, the results should be interpreted cautiously if temperature-induced sex reversal may have altered the sex-ratios obtained here [86]. Nevertheless, to the best of our knowledge, there is no reason to suspect that such phenomenon has taken place in this experiment, as it has not been described in this species.

## Conclusions

To conclude, the effects of exposure to ammonium during the larval stage on adult body size strongly depended on the temperature experienced prior to the metamorphosis. Whereas larvae reared in non-heated tanks responded to ammonium with a lower body size as adults, the opposite was true for those reared in heated tanks. This could be a consequence of temperature mediating detoxifying metabolic routes of tadpoles. Body condition was greater in ammonium-exposed individuals, in which locomotor performance was reduced, as compared with non-ammonium-exposed conspecifics. This could reflect a prioritization of the storage of resources to the detriment of locomotion in ammonium-exposed individuals, or a limitation in the ability to optimize locomotion but not storage of resources. These effects could lead to interpopulation differences, as they are more likely to reveal themselves in areas where ammonium is pervasive, such as agrosystems. In any case, these findings contrast with the fact that, at the larval and metamorphic stages, the effects of ammonium were negligible and potentially masked by the overriding consequences of high temperature, which gave rise to larger tadpoles that metamorphosed faster and into smaller metamorphs as compared with those reared in non-heated tanks.

## Supplementary Information

Supplementary file 1.

## Data Availability

The datasets used and analyzed during the current study are available from the corresponding author on reasonable request.
