# Supplementary material for: When time turns the tide: the interactive effects of ammonium and warming during the larval stage on the resulting adult frogs
Source: Front Zool. 2025 Nov 13;22:34. doi: 10.1186/s12983-025-00585-z (PMC12613779; doi:10.1186/s12983-025-00585-z)
Supplement: Supplementary file 1 [file 12983_2025_585_MOESM1_ESM.doc]

**When time turns the tide: the interactive effects of ammonium and warming during the larval stage on the resulting adult frogs**

**SUPPLEMENTARY MATERIAL**

Table S1 – Values (mean ± standard error) of temperature (°C; Table S1a) and ammonium concentration (mg/L; Table S1b) in the aquaria where the frogs were reared during their larval stages. Measurements were made twice a week during the larval stage, until the first metamorphs started to emerge. Temperature was significantly higher in heated aquaria, and ammonium concentration was significantly greater in ammonium-supplemented aquaria (results published elsewhere: reference [57] in the main text).

Table S1a

| Measurement | Heated, supplemented tanks | Non-heated, supplemented tanks | Heated, non-supplemented tanks | Non-heated, non-supplemented tanks |
| --- | --- | --- | --- | --- |
| 1 | 25.26 ± 0.26 | 20.84 ± 0.26 | 25.38 ± 0.26 | 20.86 ± 0.26 |
| 2 | 26.06± 0.26 | 22.99 ± 0.26 | 25.96 ± 0.26 | 23.01 ± 0.26 |
| 3 | 26.16± 0.26 | 22.21 ± 0.26 | 25.45 ± 0.26 | 22.30 ± 0.26 |
| 4 | 26.50± 0.26 | 22.70 ± 0.26 | 26.39 ± 0.26 | 22.28 ± 0.26 |
| 5 | 27.18± 0.26 | 23.75 ± 0.26 | 27.53 ± 0.26 | 23.89 ± 0.26 |
| 6 | 27.03± 0.26 | 22.06 ± 0.26 | 26.84 ± 0.26 | 22.01 ± 0.26 |
| 7 | 27.94± 0.26 | 24.09 ± 0.26 | 27.95 ± 0.26 | 24.16 ± 0.26 |
| 8 | 28.01± 0.26 | 24.61 ± 0.26 | 27.50 ± 0.26 | 24.81 ± 0.26 |
| 9 | 27.69± 0.26 | 24.59 ± 0.26 | 27.95 ± 0.26 | 24.68 ± 0.26 |
| 10 | 27.49± 0.27 | 23.35 ± 0.26 | 27.23 ± 0.26 | 23.41 ± 0.26 |
| 11 | 28.51± 0.27 | 25.93 ± 0.26 | 28.70 ± 0.27 | 26.06 ± 0.26 |

Table S1b

| Measurement | Heated, supplemented tanks | Non-heated, supplemented tanks | Heated, non-supplemented tanks | Non-heated, non-supplemented tanks |
| --- | --- | --- | --- | --- |
| 1 | 1.00 ± 1.12 | 2.45 ± 1.12 | 0.45 ± 1.12 | 0.00 ± 1.12 |
| 2 | 6.90 ± 1.12 | 4.00 ± 1.12 | 0.00 ± 1.12 | 0.60 ± 1.12 |
| 3 | 8.05 ± 1.12 | 4.85 ± 1.12 | 0.95 ± 1.12 | 0.85 ± 1.12 |
| 4 | 3.70 ± 0.087 | 3.83 ± 0.87 | 1.10 ± 0.087 | 0.53 ± 0.87 |
| 5 | 2.60 ± 0.087 | 4.20 ± 0.87 | 1.45 ± 0.087 | 1.03 ± 0.87 |
| 6 | 4.00 ± 0.087 | 3.45 ± 0.87 | 1.70 ± 0.087 | 0.70 ± 0.87 |
| 7 | 2.75 ± 0.087 | 2.68 ± 0.87 | 0.20 ± 0.087 | 1.40 ± 0.87 |
| 8 | 5.08 ± 0.087 | 8.80 ± 0.87 | 1.03 ± 0.087 | 1.20 ± 0.87 |
| 9 | 2.75 ± 0.087 | 4.50 ± 0.87 | 0.48 ± 0.087 | 1.08 ± 0.87 |
| 10 | 3.18 ± 0.087 | 9.13 ± 0.87 | 0.60 ± 0.087 | 4.13 ± 0.87 |
| 11 | 1.60 ± 0.087 | 5.08 ± 0.87 | 0.75 ± 0.087 | 1.85 ± 0.87 |

Table S2 – *P*-values of the Tukey post-hoc test applied on the three-way thermal treatment*ammonium interaction on SVL. Significant results are in bold.

|  | Thermal Treatment | Ammonium | Combination 1 | Combination 2 | Combination 3 | Combination 4 |
| --- | --- | --- | --- | --- | --- | --- |
| Combination 1 | Non-heated | With |  | **<0.001** | 0.394 | 1.000 |
| Combination 2 | Non-heated | Without | **<0.001** |  | **0.003** | **<0.001** |
| Combination 3 | Heated | With | 0.394 | **0.003** |  | 0.056 |
| Combination 4 | Heated | Without | 1.000 | **<0.001** | 0.056 |  |

Table S3 – *P*-values of the Tukey post-hoc test applied on the three-way thermal treatment*ammonium interaction on body mass. Significant results are in bold.

|  | Thermal Treatment | Ammonium | Combination 1 | Combination 2 | Combination 3 | Combination 4 |
| --- | --- | --- | --- | --- | --- | --- |
| Combination 1 | Non-heated | With |  | **<0.001** | **0.046** | 1.000 |
| Combination 2 | Non-heated | Without | **<0.001** |  | 0.610 | **<0.001** |
| Combination 3 | Heated | With | **0.046** | 0.610 |  | **<0.001** |
| Combination 4 | Heated | Without | 1.000 | **<0.001** | **<0.001** |  |
